# Discovery of Novel Potential Reversible Peptidyl Arginine Deiminase Inhibitor

**DOI:** 10.3390/ijms20092174

**Published:** 2019-05-02

**Authors:** Ardita Aliko, Marta Kamińska, Katherine Falkowski, Ewa Bielecka, Malgorzata Benedyk-Machaczka, Stanisław Malicki, Joanna Kozieł, Alicia Wong, Danuta Bryzek, Tomasz Kantyka, Piotr Mydel

**Affiliations:** 1 Broegelmann Research Laboratory, Department of Clinical Science, University of Bergen, N-5021 Bergen, Norway; ardita.aliko@uib.no; 2 Department of Microbiology, Faculty of Biochemistry, Biophysics and Biotechnology, Jagiellonian University, 30-387 Kraków, Poland; marta_kaminska@outlook.com (M.K.); kasia.falkowski@gmail.com (K.F.); joanna.koziel@uj.edu.pl (J.K.); alicia.wong@uj.edu.pl (A.W.); danuta.bryzek@uj.edu.pl (D.B.); 3 Malopolska Centre of Biotechnology, Jagiellonian University, 30-387 Kraków, Poland; ewa.bielecka@gmail.com (E.B.); malgorzata.benedyk@uj.edu.pl (M.B.-M.); stanislaw.malicki@uj.edu.pl (S.M.)

**Keywords:** PAD4, inhibitor, citrullination, rheumatoid arthritis, PPAD, NETs

## Abstract

Citrullination, a posttranslational modification, is catalyzed by peptidylarginine deiminases (PADs), a unique family of enzymes that converts peptidyl-arginine to peptidyl-citrulline. Overexpression and/or increased PAD activity is observed in rheumatoid arthritis (RA), Alzheimer’s disease, multiple sclerosis, and cancer. Moreover, bacterial PADs, such as *Porphyromonas gingivalis* PAD (PPAD), may have a role in the pathogenesis of RA, indicating PADs as promising therapeutic targets. Herein, six novel compounds were examined as potential inhibitors of human PAD4 and PPAD, and compared to an irreversible PAD inhibitor, Cl-amidine. Four of the tested compounds (compounds **2**, **3**, **4**, and **6**) exhibited a micromolar-range inhibition potency against PAD4 and no effect against PPAD in the in vitro assays. Compound **4** was able to inhibit the PAD4-induced citrullination of H3 histone with higher efficiency than Cl-amidine. In conclusion, compound **4** was highly effective and presents a promising direction in the search for novel RA treatment strategies.

## 1. Introduction

Citrullination is a post-translational modification of protein-bound arginine into the nonstandard amino acid, citrulline, catalyzed by Ca^2+^-dependent peptidylarginine deiminase (PAD) enzymes [1]. The citrullination process significantly alters the structure, stability and function of proteins and, as such, may affect numerous physiological and pathological processes, including gene expression, inflammation, or even the facilitation of metastasis [2,3,4]. To date, five isoforms of this enzyme have been identified (PAD1 to 4 and PAD6) with different tissue expression patterns and consequently different functions [5]. PAD-induced citrullination has been studied in different physiological and pathological conditions. PAD1, physiologically implicated in the keratinization of the skin, has been demonstrated to be hypofunctional in psoriasis; on the other hand, PAD2, essential for myelin sheath stability and the plasticity of the brain, is hyperfunctional in multiple sclerosis. PAD4, the most prominent among PADs, plays a role in tumorigenesis by influencing the expression of p53 target genes and is strongly implicated in the pathogenesis of atherosclerosis by modulating of the function of chemokines, antibacterial neutrophil extracellular traps (NETs) formation and in the generation of new autoantigens in rheumatoid arthritis (RA) [5]. Autoantibodies against citrullinated proteins (ACPAs) are detectable in about 70% of patients with RA, can be present years before the clinical onset and became an important prognostic biomarker [6]. As citrullinated proteins are highly specific targets of the immune response in RA, it had been suggested that dysregulated PAD activity is a hallmark of the immune tolerance breach driving the autoimmunity in RA. Indeed, both PAD2 and PAD4 are detected in RA synovium and are closely associated with neutrophil infiltration of synovial tissues and the intensity of inflammation [7]. Curiously, PAD4 polymorphism is associated with RA susceptibility in Asian and North American populations [8,9,10], further strengthening its relevance to the pathogenesis of this disease.

Moreover, PAD4 activity links citrullination, inflammation and autoimmunity by its role in the formation of neutrophil extracellular traps (NETs). During a type of cell death, known as NETosis, neutrophils release a highly decondensed chromatin meshwork of DNA, histones and a number of other various proteins and peptides [11] that are typically present within the neutrophil granules. Although considered a host defense mechanism, excessive NET formation contributes to the development of many noninfectious diseases, including RA, systemic lupus erythematous, diabetes, atherosclerosis, sepsis, thrombosis and even cancer [12]. PAD4 is essential for the formation of NETs by promoting chromatin decondensation through histone citrullination [13,14], mainly H3 [15,16]. Enhanced NETosis was observed in neutrophils isolated from blood and synovial fluid of patients with RA, when compared to the neutrophils from healthy controls [17]. Moreover, citrullinated histones H2A, H2B [18], H3 [19], and H4 [20] have been described as targets of ACPA antibodies generated by patients with RA.

In addition to the human PAD2 and PAD4, evidence suggests that bacterial PAD also may play a role in the pathogenesis of RA. Among prokaryotes several pathogenic *Porphyromonas* species, including *Porphyromonas gingivalis*, produce peptidylarginine deiminase (PPAD), that in contrast to mammalian PADs, predominantly deiminates C-terminal arginine in both bacterial and host proteins [21,22], thus generating novel citrullinated epitopes. *P. gingivalis* is considered a keystone pathogen in periodontitis, a disease similar to RA which is characterized by chronic self-sustaining inflammation [23]. The PPAD activity of this species could be responsible for the clinical association observed between RA and periodontitis [24]. Hence, both human PADs and their bacterial counterpart may present a possible therapeutic target in the treatment of RA.

Given the fundamental role PAD4 has in the transcriptional regulation and RA pathogenesis, plenty of research and development work has attempted to develop PAD4 inhibitors to regulate PAD4 activity and facilitate RA regression [25,26,27]. In recent years, several PAD inhibitors have been described, but most of these compounds are relatively inefficient [28,29]. For now, the most effective inhibitors are the irreversible haloacetamidine compounds, e.g., F- and Cl-amidine, with IC_50_ ranging from 1.9 to 22 μM [26]. Both F- and Cl-amidine have been shown to be active against PAD4 in vitro and in vivo [25,26]. Cl-amidine has been shown to reduce clinical signs and symptoms of colitis [30] and a decrease in the severity of murine collagen-induced arthritis (CIA) [31]. One of the second generation PAD inhibitors, BB-Cl-amidine, ameliorates the severity of joint inflammation in CIA mice through modulation of the T-cell immune responses [32]. Additionally, Wang and co-workers developed compound YW3-56, a Cl-amidine analog with improved bioavailability [33]. This compound was able to alter *SESN2* which encodes an upstream inhibitor of the mammalian target of rapamycin complex 1 (mTORC1) signaling pathway, illustrating its potential to target anticancer reagents [33]. Most of the reversible inhibitors including taxol, streptomycin and minocycline are relatively weak with millimolar IC_50_ values [25], except GSK199 and GSK484 [14], the first highly potent PAD4-specific inhibitors with IC_50_ of 0.2 and 0.05 μM. Although the number of available PAD4 inhibitors increased dramatically in recently years, they are still far from a mechanism-based drug for PAD4. Therefore, developing novel and highly potent PAD4-specific inhibitors is crucial. In this study, six novel compounds were examined as potential PAD4 and PPAD inhibitors in comparison to the widely used irreversible inhibitor, Cl-amidine.

## 2. Results

### 2.1. GST-PAD4 and HisTag-PPAD Kinetics

**Figure 1:**
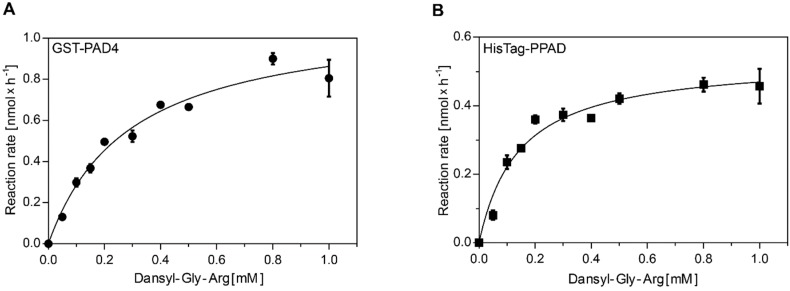
Michaelis–Menten enzyme kinetics for GST-PAD4 (**A**) and HisTag-PPAD (**B**). The rate of enzymatic product formation was plotted against the initial substrate concentration. Reaction rates were determined by incubating GST-PAD4 or PPAD with increasing concentrations of Dansyl-Gly-Arg in the presence of 10 mM CaCl_2_ and 10 mM DTT for 60 min prior to product separation by HPLC. Amount of the product formation was calculated based on the product peak integration, compared to the calibration curve. Experimental data (circles, GST-PAD; squares, PPAD), as well as the fitted model (curves) are shown. Results are expressed as mean ± SD, with *n* = 3.

The kinetic parameters of the arginine-citrulline conversion were determined by the measurement of the conversion rate in the series of increasing Dansyl-Gly-Arg substrate concentrations. Reaction products were separated by HPLC and the resulting chromatograms were analyzed by peak integration and standard curve calculation. The data was fit to the Michaelis-Menten equation and resulted in the K_m_ of 290 and 14 µM and k_cat_ 0.46 and 0.81 s^−1^ for GST-PAD4 and HisTag-PPAD, respectively (Figure 1). Calculated k_cat_ values are based on the protein concentration in the sample, not on the enzyme titration and, as such, should be regarded as the minimal values, assuming fully active enzymes. The k_cat_/K_m_ estimates are 1.5 × 10^3^ for GST-PAD4 and 5.7 × 10^4^ for HisTag-PPAD, corresponding with the previously described substrate preference of these enzymes [21,34].

### 2.2. Analysis of GST-PAD4 and HisTag-PPAD Inhibition by Compounds **1**–**6**

**Figure 2:**
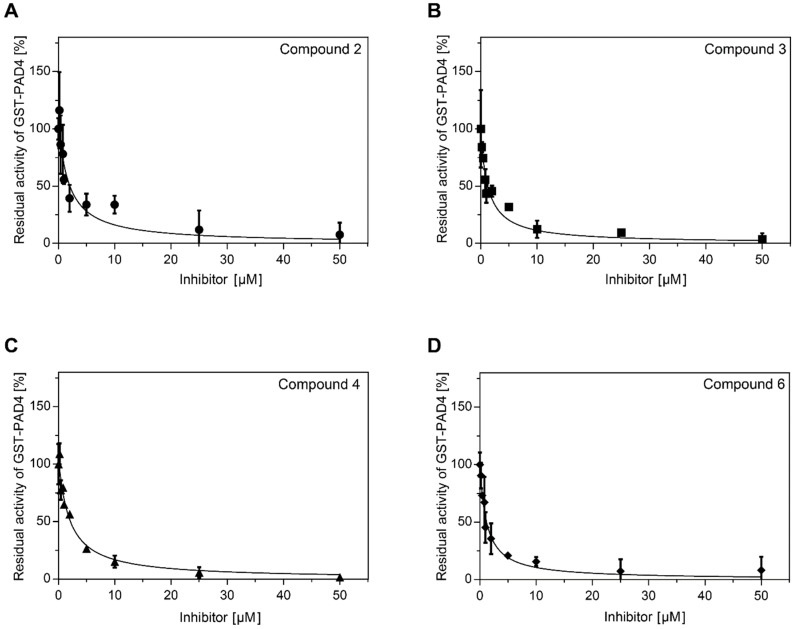
Inhibition of GST-PAD4 citrullination activity by compound **2** (**A**), **3** (**B**), **4** (**C**), and **6** (**D**). The residual activity of GST-PAD4 was plotted against the inhibitor compound concentration. Reaction rates were determined by pre-incubating GST-PAD4 and each compound in the presence of 10 mM CaCl_2_ and 0.5 mM DTT for 30 min prior to the addition of 0.25 mM Dansyl-Gly-Arg to initiate the enzyme assay. Individual samples were analyzed by HPLC and product amount was estimated based on the peak area and compared to the GST-PAD4-released product in the same reaction buffer in the absence of the compounds. Experimental data (circles, compound **2**; squares, compound **3**; triangles, compound **4**, and diamonds, compound **6**) as well as the fitted model (curves) are shown. Results are expressed as mean ± SD, with *n* = 3.

Initial screening of the compounds **1**–**6** was performed in the range of 0–250 µM inhibitor and constant 0.25 mM Dansyl-Gly-Arg substrate concentrations. GST-PAD4 was partially inhibited by compounds **2**, **3**, **4**, and **6**, while no inhibition by compounds **1** and **5** was observed. No inhibition of HisTag-PPAD was observed for any of the tested inhibitors. Further, the inhibition assay was repeated at the lower concentrations of reducing agent in the assay buffer (DTT 0–10 mM, data not shown) and increased potential of compounds **2**, **3**, **4**, and **6** was noted. The optimal inhibition was observed for the 0.5 mM DTT concentration (data not shown) and these conditions were chosen for the subsequent experiments. The values of K_i_ and IC_50_ for the tested compounds were determined in the series of inhibitor concentrations (Table 1). K_i_ were determined by the nonlinear fit of the Morrison equation to the experimental data from the range of inhibitor concentrations, while IC_50_ was defined as the inhibitor concentration at which reaction rate reaches 50% of the maximum and determined by the linear regression for each inhibitor (Figure 2). The determined parameters indicate a good affinity of the compounds **2**, **3**, **4**, and **6** to the GST-PAD4, in the low µM range for all compounds when tested in the optimized in vitro conditions, and the 50 µM concentration was chosen for subsequent experiments. However, compounds **1** and **5** exhibited no effect on the GST-PAD4 activity, and were therefore excluded from further study.

Physiological conditions cannot be fully defined and controlled, especially regarding the interacting proteins, calcium concentrations and the reducing potential in the disease-affected areas, therefore we aimed to evaluate the compound potency in the samples collected from rheumatoid arthritis patients.

**Figure 3:**
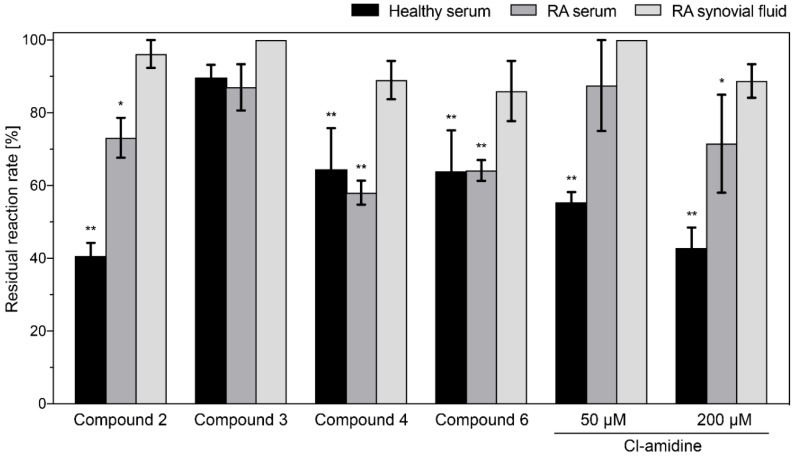
Residual activity of GST-PAD4 after inhibition by candidate compounds and Cl-amidine in the presence of serum from healthy blood donors, serum or synovial fluid from patients with RA. GST-PAD4 was pre-incubated with DMSO alone, 50 μM of candidate compounds or 50 μM and 200 μM of Cl-amidine in the presence of 10 mM CaCl_2_, 0.5 mM DTT and 1:3 diluted serum or synovial fluid for 30 min prior to the addition of 1 mM Dansyl-Gly-Arg to initiate the enzyme assay. Product generation was evaluated by HPLC. Residual activity was determined in comparison to the respective GST-PAD4 control preincubated with DMSO alone. Black bars represent healthy serum, mid gray RA serum and light grey RA synovial fluid, respectively. Results are expressed as mean ± SD, with *n* ≥ 3, and the differences were considered significant if (*) *p* < 0.05 or (**) *p* ≤ 0.01.

First, the optimal serum dilution for GST-PAD4 activity in activity assay was determined. Serum dilutions in the range 1:1.5–1:30 were tested. Results indicated that the highest GST-PAD4 activity was achieved in the serum diluted four or more times, therefore, the 1:3 dilution was chosen for the subsequent experiments. Next, the activity of the 50 µM inhibitory compounds on GST-PAD4 in the presence of serum of healthy donors, as well as the serum and synovial fluid of patients with RA, all diluted 1:3 in the assay buffer, was examined. The standard inhibitor, Cl-amidine, was employed as a positive control in 50 and 200 µM concentration and compared to the tested compounds under the same experimental conditions. The candidate molecules 1 and 5 did not show any detectable inhibitory activity when tested in the presence of healthy controls or RA-patients serum, nor in patients synovial fluid, indicating a significant loss of potential of these compounds in the in vivo-mimicking conditions (Figure 3), whereas compound **3** retained weak inhibitory activity in the presence of serum from healthy donors. Curiously, compounds **2**, **4**, and **6** retained their inhibitory potential in the presence of serum, however it was diminished when compared to the in vitro conditions. No compound remained active in the presence of RA synovial fluid. Interestingly, Cl-amidine, a standard, nonselective inhibitor of the PAD family of enzymes, showed similar behavior, partially losing its inhibitory potential in the presence of serum and becoming ineffective in the presence of RA synovial fluid. Notably, none of the inhibitors reduced the GST-PAD4 activity by more than 60% in the presence of serum or synovial fluid (Figure 3). Significantly, unlike all other compounds, inhibitory activity of compounds **4** and **6**, although affected by the presence of serum from healthy donors, did not diminish further in the presence of serum from RA-patients, indicating improved stability of these compounds in in vivo-mimicking conditions when compared to the Cl-amidine.

### 2.3. Inhibition of Histone H3 Citrullination and NET Formation

**Figure 4:**
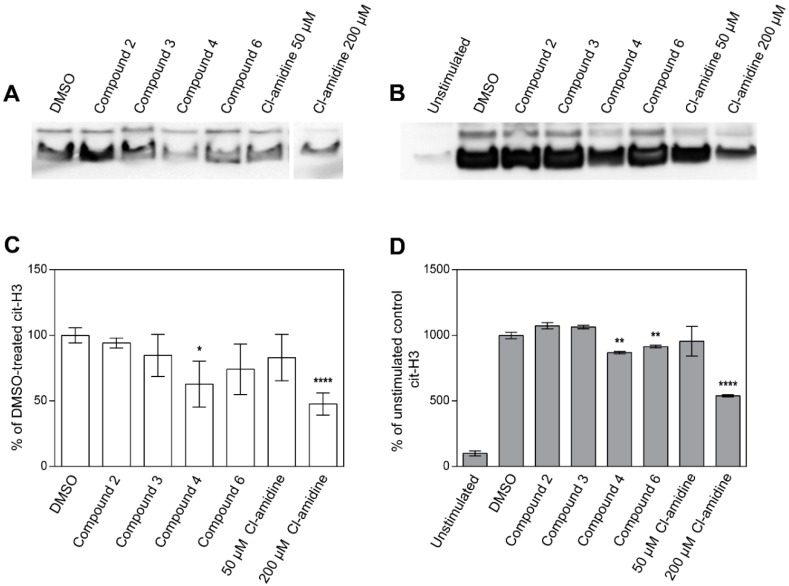
Inhibition of histone H3 citrullination in vitro (**A**,**C**) and in isolated human neutrophils (**B**,**D**). (**A**) GST-PAD4 was pre-incubated with DMSO alone, 50 μM of candidate compounds or 50 μM and 200 μM of Cl-amidine in the presence of 10 mM CaCl_2_ and 0.5 mM DTT for 30 min prior to the addition of 5 μg of purified calf thymus histone H3 to initiate the enzyme assay. Following an incubation period of 60 min, protein concentration was normalized and histones were analyzed by Western blotting with an antibody against citrullinated histone H3. A representative blot of three experiments is shown. Compounds **1** and **5** are not shown in the figure. (**B**) Human neutrophils, isolated from healthy blood donors, were pre-incubated with DMSO alone, 50 μM of candidate compounds or 50 and 200 μM of Cl-amidine and then stimulated with 4 μM calcium ionophore. Total histones were acid-extracted, protein concentration normalized and analyzed by Western blotting with an antibody against citrullinated histone H3. Representative blots of three independent experiments are shown. Band intensity was analyzed using ImageJ (freeware developed by NIH, Bethesda, USA) and values were compared to the DMSO-only treated control with the Student t-test and the differences were considered significant if (*) *p* < 0.05, (**) *p* ≤ 0.01 or (****) *p* ≤ 0.0001. Graphs represent inhibition of the histone H3 citrullination in vitro (**C**) and in stimulated neutrophils (**D**).

In order to follow the effect of the identified inhibitory compounds on the previously reported biological activity of PAD4 [16], their ability to inhibit GST-PAD4-mediated citrullination of histone H3 was verified. Purified calf thymus histone H3 was incubated with GST-PAD4 in the presence of 50 µM of the tested compounds, two concentrations of Cl-amidine, or in the absence of PAD4 inhibitors, and the citrullination was evaluated by western blot (Figure 4A,C). Only compound **4** significantly hampered the histone citrullination, inhibiting the reaction at 50 µM concentration, to the extent comparable to 200 µM Cl-amidine, thus indicating its superior effectiveness. All other compounds and 50 µM Cl-amidine were not effective, as no significant change in the citrullinated histone H3-specific band could be observed.

Histone H3 citrullination is an essential step in the neutrophil NETosis and is typically initiated by the cytoplasm calcium influx stimulated by the external signaling pathways. Therefore, the peripheral blood-isolated neutrophils were stimulated with calcium ionophore to induce Ca^2+^ downstream effects. The histone H3 citrullination was evaluated by western blot, as described above, and the inhibitory capability of compounds was estimated by comparing the band intensity to the ones resulting from neutrophil incubation with Cl-amidine (50 and 200 µM) and to the DMSO-only vehicle sample. Citrullination of histone H3 was visibly limited by 50 µM compound **4**, with efficiency higher than the 50 µM Cl-amidine, once more indicating the increased relative effectiveness of compound **4** when pitched against Cl-amidine (Figure 4B,D). Notably, compound **6** was also able to significantly inhibit the histone H3 citrullination. The compounds **2** and **3** were ineffective and resulted in the band intensity comparable to the DMSO-treated control.

**Figure 5:**
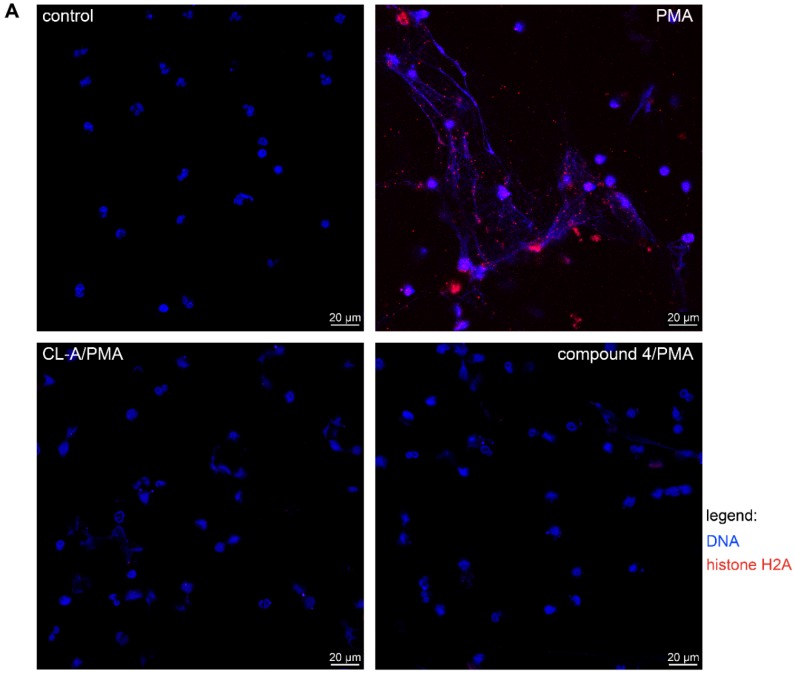

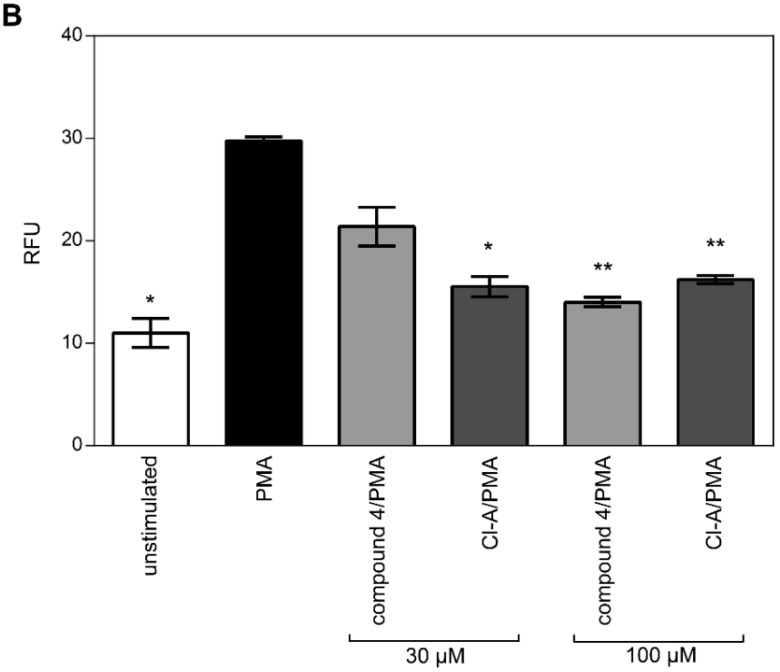
Inhibition of PAD-dependent NETs formation and release. (**A**) Human neutrophils isolated from healthy blood donors were allowed to adhere to coverslips pre-coated with poly-L-lysine. They were pre-incubated with DMSO alone, or with 100 μM of compound **4** or Cl-amidine and then stimulated with PMA. Immunostaining for histone H2 was performed with allophycocyanin (APC) F(ab’)_2_ goat anti-rabbit antibodies against histone H2A (red) with DNA counterstained with Hoechst 33258 (blue). Representative immunofluorescent images of 2 independent experiments are shown. (**B**) Human neutrophils isolated from healthy blood donors were allowed to adhere to a 24-wells plate coated with poly-L-lysine. They were pre-incubated with DMSO alone, or with either 30 μM or 100 μM of compound **4** or Cl-amidine and then stimulated with PMA. Supernatants were collected and quantification of extracellular DNA release was performed with the DNA-intercalating dye, Picogreen. Results are expressed as mean ± SD, with *n* ≥ 3 and the differences were considered significant if (*) *p* < 0.05 or (**) *p* ≤ 0.01. RFU—relative fluorescence units.

PAD4 is instrumental in the innate immunity system by the induction of NETs, the neutrophil-derived DNA expulsion with antimicrobial and inflammatory properties. NETs release was stimulated by the incubation of the peripheral blood-isolated neutrophils with PMA (phorbol 12-myristate 13-acetate) in the cells preincubated with the 30–100 µM compound **4**, Cl-amidine or DMSO as vehicle control. Expectedly, PMA incubation stimulated the neutrophils to release DNA in the inhibitor-untreated cells and 30 µM Cl-amidine inhibited this effect. Compound **4** at 30 µM displayed limited ability to block DNA release, while at the 100 µM, compound **4** was more effective than Cl-amidine (Figure 5B). Furthermore, when NET release was visualized by DNA (blue) and a specific NET marker, histone fragment H2A (red) immunostaining, only PMA-alone stimulated cells were able to release fully developed NETs, while 100 µM Cl-amidine and compound **4** blocked the NET formation (Figure 5A).

Taken together, the presented data indicate compound **4**, a commercially-available molecule, as the most effective inhibitor of GST-PAD4, potent in the in vitro biochemical assays as well in the cell-based models of PAD activity. The effectiveness of the novel compound **4** was comparable or even better than widely used Cl-amidine in all performed experiments, suggesting the compound **4** as a candidate for further development of PAD4-specific inhibitors of increased efficiency.

## 3. Discussion

Given PADs fundamental role in transcriptional regulation and aetiopathology of various inflammatory diseases, plenty of research and development work has attended to develop efficient and safe PAD inhibitors for clinical use [35]. In the present study, six novel compounds discovered using in silico high-throughput screening approach; two of them commercially available and four proprietary of 4SC AG; were assessed and compared to the well-established pan-PAD-inhibitor, Cl-amidine, for potential inhibition of PAD4 and PPAD.

Apart from the human PADs, a growing body of evidence supports a role for bacterial PAD in the initiation and pathogenesis of RA [24], thus making *P. gingivalis* PPAD a viable target for development of therapeutic inhibitors. The efforts to develop an efficient PPAD inhibitor have been unsuccessful so far and similarly, the results of our study show that none of the candidate compounds exerts activity against PPAD. This is not surprising and likely reflects the significant differences between PAD4 and PPAD in both structure and the mechanism of action. Indeed, PPAD and human PADs have a low sequence homology [36] and distinct substrate specificities [37], confirmed herein by determination of the Dansyl-Gly-Arg processing kinetics. Calculated K_m_, k_cat_ and resulting k_cat_/K_m_ values indicate nearly 40× better processing of the Dansyl-Gly-Arg substrate by bacterial PPAD, mainly a result of the higher substrate affinity. Unlike its eukaryotic counterparts, PPAD preferentially citrullinates C-terminal arginines of peptides [21] and acts in a calcium-independent manner [38]. This suggests that the common inhibitor of both enzymes will be difficult to develop, at the same time indicating the possibility that the future PPAD specific inhibitor will exhibit high selectivity and low side effects.

Our biochemical assays clearly indicate that four of the tested compounds (compound **2**, **3**, **4**, and **6**) were effective PAD4 inhibitors in the micromolar range. These compounds showed around 3–5-fold increase in PAD4 inhibition when pitched against Cl-amidine (IC_50_ = about 1.28–1.88 μM, compared to Cl-amidine with IC_50_ = about 5 μM) [33], signifying the improved effectiveness of the inhibition and providing basics for the future targeted drug development and inhibitor design. Similarly, the presented data indicate the importance of the host derived factors in the development of inhibitors with potential therapeutic role. In experiments where serum of either healthy or patients with RA was included in the sample preparation, the effectiveness of all inhibitors, including Cl-amidine, was clearly impaired and the addition of RA synovial fluid nearly completely blocked the effect of all the inhibitors. This deleterious effects of serum and synovial fluid on the compounds’ ability to act as PAD4 inhibitors can be explained by their direct interactions with serum or synovial fluid components, e.g., binding to other proteins and/or inactivation by other enzymes present in the milieu. Alternatively, serum and synovial fluid might affect the activity of the PAD4 itself, or both effects could play a role. In RA, the synovial joint inflammation is a hallmark of the disease and prominent accumulation of citrullinated proteins and ACPAs is observed in the affected areas [39,40]. PAD4 is expressed in the synovial tissue of patients with RA and is believed to play an essential role in the generation of synovial ACPA targets. Therefore, targeting PAD4 in synovium is a valid strategy of protection from the autoimmune reaction and the local interactions need to be included in the analysis of the inhibitor effectiveness.

Previous studies have correlated PAD4 expression with the histone citrullination [13,14] and identified histone H3 as the preferred substrate for PAD4 [15,16]. As such, PAD4 has an essential role in the chromatin decondensation and subsequent NET formation, thus contributing to the development of many inflammatory-related diseases, including RA [12]. Therefore, the inhibitory effects of the investigated molecules on histone H3 citrullination and NET formation were determined in the current study. Purified histone H3 and cell culture data were compatible and identified compound **4** as a novel inhibitor of PAD4-mediated histone H3 citrullination. Again, the citrullination of the histone H3 was inhibited more efficiently by compound **4** when compared to the Cl-amidine. In contrast, 30 µM Cl-amidine was superior in the NET formation in vitro cell assay, in comparison to the compound **4** at the same concentration. Nonetheless, both inhibitors were equally effective at 100 µM concentration and the observed differences may be explained by the membrane permeability of the inhibitor, a property likely to be optimized in the design process.

Selective PAD4 inhibitors have been described by Lewis and co-workers [14,41], allowing to validate the critical role of PAD4 in both histone citrullination and neutrophil extracellular trap formation. Therefore, the identification of isoenzyme-specific PAD inhibitors is desirable, given the essential functions of the human PADs in normal physiology [2]. As our investigations were focused on the selection of the kinetically best candidate compound in vitro and ex vivo, the selectivity of the compounds was not evaluated.

An important consideration for the inhibitor analysis is the kinetic model of inhibition. In the presented study we assumed a tight-binding competitive inhibition. This allows the K_i_ determination and is a good approximation of both reversible and irreversible inhibitors, where the irreversible inhibitor would be characterized by high k_on_, but k_off_ equal (or close to) zero. Nonetheless, the exosite binding and allosteric inhibition cannot be followed in this model and require further analysis. In addition to K_i_, we provide the measurement of IC_50_, as a universal parameter allowing the model-independent comparison of the compounds under the same experimental conditions. Another important consideration is the composition of the reaction buffer. Reducing agents, pH, and Ca^2+^ are essential factors regulating the PAD4 activity [42] and indeed, the DTT concentration was critical for the inhibitory effect of all compounds, including Cl-amidine. The in vitro assays were optimized to provide the correct inhibition for kinetic analysis and included 0.5 mM DDT in the reaction buffer, however, this could not be controlled when patient samples were included. Therefore, the diminished potential of the inhibitors in the serum and/or synovial fluid could be explained, at least partially, by the decreased redox potential in the biological fluids, when compared to the reaction buffer alone. Nonetheless, despite possible limitations, compound **4** was identified as an optimal inhibitor in all assays, including the cell culture in vitro analysis.

In conclusion, herein we describe four novel PAD4 inhibitor compounds. All are characterized by the micromolar-range inhibition potency against PAD4 in vitro. Similarly to Cl-amidine, they demonstrated impaired efficacy in the presence of serum and synovial fluid from patients with RA. Importantly, the best candidate (compound **4**) outperformed Cl-amidine, the commonly used pan-PAD inhibitor, in almost all assays and is freely available commercially. We believe that compound **4** presented here may serve as a design template for further optimization and will allow development of specific inhibitors allowing the investigation of the biology of PAD4 and introduction of the new strategies of RA treatment.

## 4. Materials and Methods

### 4.1. Compounds

**Figure 6:**
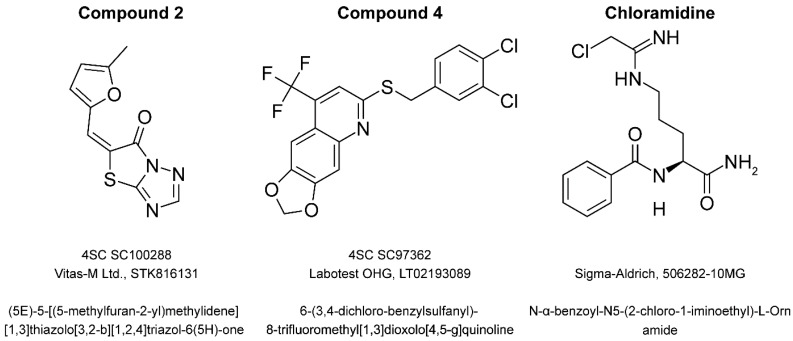
Structures of the commercially available compounds: compound **2**, **4**, and Cl-amidine. Individual compounds are identified by 4SC internal ID number and manufacturer catalog number. Chemical names are provided.

Four proprietary compounds, protected by an NDA agreement, and two commercially available molecules, as listed in Table 2, were kindly provided by 4SC AG (Planegg-Martinsried, Germany). Compounds were selected by an in silico high-throughput screening approach as potential inhibitors of peptidyl deiminase activity. The chemical structures of commercially available compounds **1** and **4** are presented in Figure 6. All compounds were dissolved in DMSO at 10 mM and stored as stock solutions at −20 °C until used.

### 4.2. Patient Samples

Sera samples and synovial fluid were collected from patients with RA and healthy controls at the Haukeland University Hospital in Bergen. The study was approved by the Regional Committee of Medical and Health Research Ethics (REK 3.2006.3085) and all participants gave informed consent. Neutrophils were isolated from peripheral venous blood samples, collected from voluntary blood donors from the blood bank at the Haukeland University Hospital in Bergen (Norway), or the Red Cross (Krakow, Poland) and was appropriately de-identified for human subjects confidentiality assurance. Therefore, this study adheres to appropriate exclusions from human subjects approval and appropriate state ethical regulations.

### 4.3. Reagents

GST-PAD4 was purified according to the modified protocol published elsewhere [43] with required changes for purification of the GST-tagged proteins. *Porphyromonas gingivalis* HisTag-PPAD was purified as described previously by Bielecka et al. [44]. Dansyl-Gly-Arg and Dansyl-Gly-Cit peptides were custom-synthetized by Thinkpeptides (Oxford, UK). Purified histone H3, isolated from calf thymus, was purchased from Roche Diagnostics (Mannheim, Germany) and dissolved in MilliQ water. Cl-amidine was purchased from Calbiochem (Darmstadt, Germany) and dissolved in DMSO. DMSO was used as vehicle control in each experiment.

### 4.4. Measurement of Enzyme Kinetics

The kinetic parameters of GST-PAD4 and HisTag-PPAD were assessed using Dansyl-Gly-Arg as substrate, with concentrations ranging from 0.05 mM to 1 mM, and Dansyl-Gly-Cit as a product standard. A total of 1 mU of each enzyme was used, which corresponds to the activity resulting in the conversion of 1 nmol of arginine into citrulline within 1 h of the reaction. The assay was carried out in 30 μL of assay buffer consisting of 100 mM Tris pH 7.5, 10 mM CaCl_2_ and 10 mM DTT at 37 °C for 60 min, the reaction was terminated by adding 5% trichloroacetic acid (TCA) and incubated on ice for 15 min. The samples were adjusted to 0.3% trifluoracetic acid (TFA) in 110 µL final volume, centrifuged, and the supernatant was analyzed via HPLC (Shimadzu Corp., Kyoto, Japan). Separation was achieved on an Aeris XB-C18 4.6/150 (Phenomenex, Torrance, CA, USA) reverse-phase column in the gradient of Phase A (0.1% TFA in water) and Phase B (80% acetonitrile, 0.08% TFA in water). Briefly, 20 μL of each sample was injected onto the column equilibrated in 10% of Phase B, the reaction products were eluted in the separating gradient 10–30% of Phase B in 25 column volumes, under the flow of 1.5 mL/min. Fluorescence emitted by dansyl (excitation at 342 nm, emission at 562 nm) was monitored and the citrullination activity of PADs was calculated from the product peak area of citrullinated Dansyl-Gly-Arg, relative to external calibration curves obtained with five different concentrations of the Dansyl-Gly-Cit standard.

### 4.5. Measurement of Inhibitor Constants and IC_50_

GST-PAD4 and HisTag-PPAD were pre-incubated for 30 min at 37 °C with DMSO alone or with increasing concentrations (0.2–250 μM) of compounds **1** to **6** in the assay buffer (100 mM Tris pH 7.5, 10 mM CaCl_2_ and DTT). 1 mU of GST-PAD4 was used in the experiments. To optimize the concentration of the reducing agent, experiments were done using different concentrations of DTT in the assay buffer ranging from 0 to 10 mM and the final concentration for the presented results was 0.5 mM DTT. Following pre-incubation of enzyme in the assay buffer, the reaction was initiated by the addition of 0.25 mM Dansyl-Gly-Arg. The reaction was stopped after 1h (as described above), the sample was separated by HPLC and the relative activity of PAD in each sample was determined by measuring the peak area of the citrullinated Dansyl-Gly-Arg.

### 4.6. Measurement of Inhibitory Activity in Serum and Synovial Fluid

A total of 50 μM of compounds **1** to **6** were pre-incubated with 1 mU of GST-PAD4 for 30 min at 37 °C with serum from healthy blood donors, serum or synovial fluid from patients with RA, serially diluted from 1:1.5 to 1:30 in the assay buffer (100 mM Tris pH 7.5, 10 mM CaCl_2_). The reaction was then initiated by the addition of 1 mM Dansyl-Gly-Arg and stopped after 1h as described above. The assay included a negative and positive control: a DMSO (no compound) control and a known irreversible compound (Cl-amidine) at 50 and 200 μM. Samples were separated by HPLC.

### 4.7. Histone H3 Citrullination Assay

A total of 50 μM of compounds **1** to **6** were pre-incubated with 1 mU of GST-PAD4 for 30 min at 37 °C in the assay buffer (100 mM Tris pH 7.5, 10 mM CaCl_2_, 0.5 mM DTT). After that, 5 μg of purified calf thymus histone H3 was added to the reaction mixture and incubated further for 60 min at 37 °C. Three controls were performed in the assay: a DMSO (no compound) control and a known irreversible compound (Cl-amidine) at 50 and 200 μM. The citrullination was assessed by western blot using antibodies specific to citrullinated histone H3.

### 4.8. Histone H3 Citrullination in Neutrophils Stimulated with Calcium Ionophore

Neutrophils were isolated from human peripheral blood collected in acid citrate dextrose (ACD-A) by first spinning blood on Lymphoprep^TM^ (Axis Shield, Oslo, Norway), followed by dextran sedimentation using 3% dextran density gradient and hypotonic lysis of erythrocytes. Neutrophil purity was assessed to be >90% by flow cytometry. Cells were resuspended at 2 × 10^6^ cells/mL in RPMI-1640 with HEPES (Thermo Fisher Scientific, Waltham, MA, USA) supplemented with 2 mM CaCl_2_ and pre-treated for 30 min with DMSO alone, 50 μM of compounds **1** to **6** or with either 50 or 200 μM of Cl-amidine. Cells were then stimulated with 4 μM calcium ionophore A23187 (Sigma-Aldrich, St. Louis, MO, USA) or left unstimulated as controls for 15 min at 37 °C, 5% CO_2_. Following stimulation, acid extraction of histones was performed according to Shechter et al. [45]. Briefly, cells were centrifuged (300× *g*, 10 min) and were resuspended in hypotonic lysis buffer (10 mM Tris pH 8.0, 1 mM KCl, 1.5 mM MgCl_2_) supplemented with 1 mM DTT, 1 mM PMSF and complete protease inhibitor cocktail (Roche Diagnostics, Mannheim, Germany) at a density of 2 × 10^6^ cells/mL then incubated on a rotator at 4 °C for 30 min. The nuclei were pelleted by spinning (10,000× g, 10 min, 4 °C), resuspended in 0.4 N H_2_SO_4_ and incubated on a rotator overnight at 4 °C. After spinning (16,000× *g*, 10 min, 4 °C), supernatants were transferred to fresh tubes. TCA was then added drop by drop to the histone solution (to the final concentration of 33%), after which the tubes were inverted several times and incubated on ice for 30 min. Pelleted histones (16,000× *g*, 10 min, 4 °C) were washed twice with ice cold acetone and air dried in for 20 min at room temperature. Histone pellets were dissolved in MilliQ water (Merck Millipore, Burlington, VT, USA) and transferred into fresh tubes. The presence of citrullination in cellular histones was analyzed by Western blot.

### 4.9. Western Blot

The protein concentration in each sample was measured (A_280nm_) and normalized post-extraction. Next, histone proteins were separated in precast Novex^TM^ 16% Tris-Glycine 1 mm minigels (Invitrogen, Carlsbad, CA, USA). After electrophoresis, proteins were transferred into nitrocellulose membranes (0.2 μm) at 4 °C (transfer buffer: 10 mM CAPS, 20% methanol, pH 11). Membranes were blocked for 1 h at room temperature with 5% milk in TBST (1× Tris Buffered Saline, pH 8.0, with 0.1% Tween 20) and then incubated overnight at 4 °C with rabbit polyclonal anti-H3Cit antibody (Abcam, ab5103, Cambridge, UK) at a 1:1,000 dilution in the blocking solution. All blots were probed with an HRP-linked goat anti-rabbit secondary antibody (Abcam, ab205718) at a 1:10,000 dilution and then developed using Clarity Western ECL substrate. The signal was recorded with ChemiDoc image analyzer (Bio-Rad Laboratories, Hercules, CA, USA).

### 4.10. NET Assays

Granulocyte-enriched fractions were harvested using a lymphocyte separation medium density gradient (PAN Biotech, Aidenbach, Germany). Neutrophils and erythrocytes were separated after 30 min of incubation with 1% polyvinyl alcohol (POCH, Gliwice, Poland). Neutrophils were collected from the upper layer and after centrifugation (280× *g*, 10 min), the residual erythrocytes were removed by lysis in water. Cells were then resuspended in serum-free DMEM without phenol red (Gibco, Waltham, MA, USA). To measure the extracellular DNA release, neutrophils were seeded at 2 × 10^6^/well in 0.01 mg/mL poly-L-lysine (Sigma-Aldrich) coated 24-well plates and centrifuged (200× *g*, 5 min). Neutrophils were treated for 1h with DMSO alone, or with either 30 μM or 100 μM of compound **4** or Cl-amidine. Cells were then stimulated with 25 nM PMA (phorbol 12-myristate 13-acetate) or left unstimulated as controls for 3 h at 37 °C, 5% CO_2_. Following stimulation, the cell medium was collected and the extracellular DNA was quantified using the Quant-iT^TM^ PicoGreen^®^ dsDNA Reagent (QPG; Invitrogen, Carlsbad, CA, USA). The 1:200 dilution of QPG in TE buffer (10 mM Tris, 1 mM EDTA, pH 7.5) and 90 μL of TE buffer was added to 10 μL of the supernatant containing the extracellular DNA and fluorescence was measured with an excitation wavelength 480 nm and emission wavelength 520 nm.

For the NETs imaging assays, neutrophils were allowed to adhere to poly-L-lysine coated coverslips (5 × 10^5^ cells/coverslip) for 30 min at 37 °C, 5% CO_2_. Neutrophils were then treated for 1 h with DMSO alone, or 100 μM of compound **4** or Cl-amidine. Neutrophils were then stimulated with 25 nM PMA or left unstimulated as controls for 3 h at 37 °C, 5% CO_2_. Neutrophils were fixed with 3.7% formaldehyde for 10 min, washed three times with PBS and blocked (5% FBS, 1% BSA, 0.05% Tween, 2 mM EDTA in PBS) for 1 h. Cells were washed and treated with 0.1% saponin (Sigma-Aldrich) in PBS for 30 min. Cells were stained first with rabbit polyclonal anti-histone H2A antibody (Abcam), and then with allophycocyanin (APC) F(ab’)_2_ goat anti-rabbit antibody (Jackson ImmunoResearch Lab Inc., Ely, UK) in the presence of 3% BSA in 0.1% saponin. Cells were counterstained with 1 µg/mL Hoechst 33258 dye (Invitrogen). Images were captured with a fluorescence microscope (Nikon Eclips Ti, Nikon, Konan, Tokyo).

### 4.11. Statistical Analysis

The experiments have been performed at least three times and the results are presented as means ± SD. One-way ANOVA with Dunnet’s post-test were used when comparing multiple groups. The differences were considered significant if *p* ≤ 0.05.

The Michaelis constant (K_m_) was calculated by fitting a Michaelis plot of initial rate of enzymatic product formation against the substrate concentration to the Michaelis–Menten equation using and represent the mean ± SD from three different determinations.

The equilibrium inhibition constant (K_i_) and IC_50_ were calculated from the fit of observed PAD activity against the respective compound concentration. Data was analyzed by the built-in Morrisson equation for tight-binding inhibition [46] and represent the mean ± SD from at least three different determinations. IC_50_ values were determined by the linear regression to the initial fragment of the inhibition curve and are representative of inhibitor concentration required for the 50% reduction in the product release during the reaction.

The statistical analysis was performed using GraphPad Prism 7 software (GraphPad Software, La Jolla, CA, USA).

## Figures and Tables

**Table 1:** K_i_ and IC_50_ values of candidate compounds.

Compound	K_i_ (μM) ^a^	IC_50_ (μM)
**1** ^b^	-	-
**2**	0.94 ± 0.31	1.40 ± 0.26
**3**	0.71 ± 0.17	1.65 ± 1.34
**4**	1.00 ± 0.16	1.88 ± 0.26
**5** ^b^	-	-
**6**	0.61 ± 0.12	1.28 ± 0.30

Values were determined by the equilibrium-state experiment, where GST-PAD4 was preincubated with the indicated compound prior to the substrate addition. The equilibrium inhibition constant (K_i_) for each compound was obtained from global fit to the tight-binding inhibition equations. IC_50_ was determined as the concentration of the inhibitor that yields half-maximal activity, as determined by linear regression. Values are mean ± S.D. with *n* = 3. ^a^ Assuming competitive reversible mechanism of inhibition. ^b^ No inhibition was observed at concentrations up to 250 μM.

**Table 2:** List of the tested compounds.

Compound	4SC Catalogue	Available Commercially	Chemical Structure	Manufacturer	Cat. No.
**1**	SC100288	Yes	Yes	Vitas-M Ltd.	STK816131
**2**	SC100449	n.a.	n.a.	n.a.	n.a.
**3**	SC101037	n.a.	n.a.	n.a.	n.a.
**4**	SC97362	Yes	Yes	Labotest OHG	LT02193089
**5**	SC99514	n.a.	n.a.	n.a.	n.a.
**6**	SC101038	n.a.	n.a.	n.a.	n.a.
Cl-amidine	-	Yes	Yes	Sigma-Aldrich	506282-10MG

Compounds were provided by 4SC AG and are identified with the 4SC ID numbers. Two of the molecules (**1** and **4**) are commercially available and their manufacturer and respective catalogue number is provided. Chemical structures are available for these compounds. Cl-amidine was used as the standard PAD inhibitor for validation purposes. n.a.—not available.

## References

[1] Bicker K.L., Thompson P.R. (2013). The protein arginine deiminases: Structure, function, inhibition, and disease. Biopolymers.

[2] Vossenaar E.R., Zendman A.J.W., Van Venrooij W.J., Pruijn G.J.M. (2003). PAD, a growing family of citrullinating enzymes: Genes, features and involvement in disease. BioEssays.

[3] Gudmann N.S., Hansen N.U.B., Jensen A.C.B., Karsdal M.A., Siebuhr A.S. (2015). Biological relevance of citrullinations: Diagnostic, prognostic and therapeutic options. Autoimmunity.

[4] Yuzhalin A.E., Gordon-Weeks A.N., Tognoli M.L., Jones K., Markelc B., Konietzny R., Fischer R., Muth A., O’Neill E., Thompson P.R. (2018). Colorectal cancer liver metastatic growth depends on PAD4-driven citrullination of the extracellular matrix. Nat. Commun..

[5] Baka Z., György B., Géher P., Buzás E.I., Falus A., Nagy G. (2012). Citrullination under physiological and pathological conditions. Joint. Bone. Spine Rev. Du Rhum..

[6] Van Venrooij W.J., van Beers J.J.B.C., Pruijn G.J.M. (2008). Anti-CCP Antibody, a Marker for the Early Detection of Rheumatoid Arthritis. Ann. N. Y. Acad. Sci..

[7] Foulquier C., Sebbag M., Clavel C., Chapuy-Regaud S., Al Badine R., Méchin M.-C., Vincent C., Nachat R., Yamada M., Takahara H. (2007). Peptidyl arginine deiminase type 2 (PAD-2) and PAD-4 but not PAD-1, PAD-3, and PAD-6 are expressed in rheumatoid arthritis synovium in close association with tissue inflammation. Arthritis Rheum..

[8] Suzuki A., Yamada R., Chang X., Tokuhiro S., Sawada T., Suzuki M., Nagasaki M., Nakayama-Hamada M., Kawaida R., Ono M. (2003). Functional haplotypes of PADI4, encoding citrullinating enzyme peptidylarginine deiminase 4, are associated with rheumatoid arthritis. Nat. Genet..

[9] Plenge R.M., Padyukov L., Remmers E.F., Purcell S., Lee A.T., Karlson E.W., Wolfe F., Kastner D.L., Alfredsson L., Altshuler D. (2005). Replication of putative candidate-gene associations with rheumatoid arthritis in >4,000 samples from North America and Sweden: Association of susceptibility with PTPN22, CTLA4, and PADI4. Am. J. Hum. Genet..

[10] Kang C.P., Lee H.-S., Ju H., Cho H., Kang C., Bae S.-C. (2006). A functional haplotype of the PADI4 gene associated with increased rheumatoid arthritis susceptibility in Koreans. Arthritis Rheum..

[11] Brinkmann V., Reichard U., Goosmann C., Fauler B., Uhlemann Y., Weiss D.S., Weinrauch Y., Zychlinsky A. (2004). Neutrophil extracellular traps kill bacteria. Science.

[12] Jorch S.K., Kubes P. (2017). An emerging role for neutrophil extracellular traps in noninfectious disease. Nat. Med..

[13] Li P., Li M., Lindberg M.R., Kennett M.J., Xiong N., Wang Y. (2010). PAD4 is essential for antibacterial innate immunity mediated by neutrophil extracellular traps. J. Exp. Med..

[14] Lewis H.D., Liddle J., Coote J.E., Atkinson S.J., Barker M.D., Bax B.D., Bicker K.L., Bingham R.P., Campbell M., Chen Y.H. (2015). Inhibition of PAD4 activity is sufficient to disrupt mouse and human NET formation. Nat. Chem. Biol..

[15] Cuthbert G.L., Daujat S., Snowden A.W., Erdjument-Bromage H., Hagiwara T., Yamada M., Schneider R., Gregory P.D., Tempst P., Bannister A.J. (2004). Histone deimination antagonizes arginine methylation. Cell.

[16] Darrah E., Rosen A., Giles J.T., Andrade F. (2012). Peptidylarginine deiminase 2, 3 and 4 have distinct specificities against cellular substrates: Novel insights into autoantigen selection in rheumatoid arthritis. Ann. Rheum. Dis..

[17] Khandpur R., Carmona-Rivera C., Vivekanandan-Giri A., Gizinski A., Yalavarthi S., Knight J.S., Friday S., Li S., Patel R.M., Subramanian V. (2013). NETs are a source of citrullinated autoantigens and stimulate inflammatory responses in rheumatoid arthritis. Sci. Transl. Med..

[18] Corsiero E., Bombardieri M., Carlotti E., Pratesi F., Robinson W., Migliorini P., Pitzalis C. (2016). Single cell cloning and recombinant monoclonal antibodies generation from RA synovial B cells reveal frequent targeting of citrullinated histones of NETs. Ann. Rheum. Dis..

[19] Janssen K.M.J., de Smit M.J., Withaar C., Brouwer E., van Winkelhoff A.J., Vissink A., Westra J. (2017). Autoantibodies against citrullinated histone H3 in rheumatoid arthritis and periodontitis patients. J. Clin. Periodontol..

[20] Pratesi F., Dioni I., Tommasi C., Alcaro M.C., Paolini I., Barbetti F., Boscaro F., Panza F., Puxeddu I., Rovero P. (2014). Antibodies from patients with rheumatoid arthritis target citrullinated histone 4 contained in neutrophils extracellular traps. Ann. Rheum. Dis..

[21] McGraw W.T., Potempa J., Farley D., Travis J. (1999). Purification, characterization, and sequence analysis of a potential virulence factor from Porphyromonas gingivalis, peptidylarginine deiminase. Infect. Immun..

[22] Gabarrini G., Chlebowicz M.A., Vega Quiroz M.E., Veloo A.C.M., Rossen J.W.A., Harmsen H.J.M., Laine M.L., van Dijl J.M., van Winkelhoff A.J. (2018). Conserved Citrullinating Exoenzymes in Porphyromonas Species. J. Dent. Res..

[23] Janssen K.M.J., Vissink A., de Smit M.J., Westra J., Brouwer E. (2013). Lessons to be learned from periodontitis. Curr. Opin. Rheumatol..

[24] Potempa J., Mydel P., Koziel J. (2017). The case for periodontitis in the pathogenesis of rheumatoid arthritis. Nat. Rev. Rheumatol..

[25] Knuckley B., Luo Y., Thompson P.R. (2008). Profiling Protein Arginine Deiminase 4 (PAD4): A novel screen to identify PAD4 inhibitors. Bioorg. Med. Chem..

[26] Luo Y., Knuckley B., Lee Y.-H., Stallcup M.R., Thompson P.R. (2006). A fluoroacetamidine-based inactivator of protein arginine deiminase 4: Design, synthesis, and in vitro and in vivo evaluation. J. Am. Chem. Soc..

[27] Jones J.E., Slack J.L., Fang P., Zhang X., Subramanian V., Causey C.P., Coonrod S.A., Guo M., Thompson P.R. (2012). Synthesis and screening of a haloacetamidine containing library to identify PAD4 selective inhibitors. ACS Chem. Biol..

[28] Hidaka Y., Hagiwara T., Yamada M. (2005). Methylation of the guanidino group of arginine residues prevents citrullination by peptidylarginine deiminase IV. FEBS Lett..

[29] Pritzker L.B., Moscarello M.A. (1998). A novel microtubule independent effect of paclitaxel: The inhibition of peptidylarginine deiminase from bovine brain. Biochim. Biophys. Acta.

[30] Chumanevich A.A., Causey C.P., Knuckley B.A., Jones J.E., Poudyal D., Chumanevich A.P., Davis T., Matesic L.E., Thompson P.R., Hofseth L.J. (2011). Suppression of colitis in mice by Cl-amidine: A novel peptidylarginine deiminase inhibitor. Am. J. Physiol. Liver Physiol..

[31] Willis V.C., Gizinski A.M., Banda N.K., Causey C.P., Knuckley B., Cordova K.N., Luo Y., Levitt B., Glogowska M., Chandra P. (2011). N-α-Benzoyl-N5-(2-Chloro-1-Iminoethyl)-l-Ornithine Amide, a Protein Arginine Deiminase Inhibitor, Reduces the Severity of Murine Collagen-Induced Arthritis. J. Immunol..

[32] Kawalkowska J., Quirke A.M., Ghari F., Davis S., Subramanian V., Thompson P.R., Williams R.O., Fischer R., La Thangue N.B., Venables P.J. (2016). Abrogation of collagen-induced arthritis by a peptidyl arginine deiminase inhibitor is associated with modulation of T cell-mediated immune responses. Sci. Rep..

[33] Wang Y., Li P., Wang S., Hu J., Chen X.A., Wu J., Fisher M., Oshaben K., Zhao N., Gu Y. (2012). Anticancer peptidylarginine deiminase (PAD) inhibitors regulate the autophagy flux and the mammalian target of rapamycin complex 1 activity. J. Biol. Chem..

[34] Knuckley B., Causey C.P., Jones J.E., Bhatia M., Christina J., Osborne T., Takahara H., Thompson P.R. (2010). Substrate specificity and kinetic studies of PADs 1, 3, and 4 identify potent and selective inhibitors of Protein Arginine Deiminase 3. Biochemistry.

[35] Wang S., Wang Y. (2013). Peptidylarginine deiminases in citrullination, gene regulation, health and pathogenesis. Biochim. Biophys. Acta.

[36] Shirai H., Blundell T.L., Mizuguchi K. (2001). A novel superfamily of enzymes that catalyze the modification of guanidino groups. Trends Biochem. Sci..

[37] Montgomery A.B., Kopec J., Shrestha L., Thezenas M.-L., Burgess-Brown N.A., Fischer R., Yue W.W., Venables P.J. (2016). Crystal structure of Porphyromonas gingivalis peptidylarginine deiminase: Implications for autoimmunity in rheumatoid arthritis. Ann. Rheum. Dis..

[38] Abdullah S.N., Farmer E.A., Spargo L., Logan R., Gully N. (2013). Porphyromonas gingivalis peptidylarginine deiminase substrate specificity. Anaerobe.

[39] Vossenaar E.R., Nijenhuis S., Helsen M.M.A., van der Heijden A., Senshu T., van den Berg W.B., van Venrooij W.J., Joosten L.A.B. (2003). Citrullination of synovial proteins in murine models of rheumatoid arthritis. Arthritis Rheum..

[40] Chang X., Yamada R., Suzuki A., Sawada T., Yoshino S., Tokuhiro S., Yamamoto K. (2005). Localization of peptidylarginine deiminase 4 (PADI4) and citrullinated protein in synovial tissue of rheumatoid arthritis. Rheumatology (Oxford).

[41] Willis V.C., Banda N.K., Cordova K.N., Chandra P.E., Robinson W.H., Cooper D.C., Lugo D., Mehta G., Taylor S., Tak P.P. (2017). Protein arginine deiminase 4 inhibition is sufficient for the amelioration of collagen-induced arthritis. Clin. Exp. Immunol..

[42] Jones J.E., Causey C.P., Knuckley B., Slack-Noyes J.L., Thompson P.R. (2009). Protein arginine deiminase 4 (PAD4): Current understanding and future therapeutic potential. Curr. Opin. Drug Discov. Devel..

[43] Horikoshi N., Tachiwana H., Saito K., Osakabe A., Sato M., Yamada M., Akashi S., Nishimura Y., Kagawa W., Kurumizaka H. (2011). Structural and biochemical analyses of the human PAD4 variant encoded by a functional haplotype gene. Acta Crystallogr. Sect. D Biol. Crystallogr..

[44] Bielecka E., Scavenius C., Kantyka T., Jusko M., Mizgalska D., Szmigielski B., Potempa B., Enghild J.J., Prossnitz E.R., Blom A.M. (2014). Peptidyl arginine deiminase from porphyromonas gingivalis abolishes anaphylatoxin C5a activity. J. Biol. Chem..

[45] Shechter D., Dormann H.L., Allis C.D., Hake S.B. (2007). Extraction, purification and analysis of histones. Nat. Protoc..

[46] Copeland R.A. (2000). Enzymes-A Practical Introduction to Structure, Mechanism and Data Analysis.

